# Population Interest in Information on Obesity, Nutrition, and Occupational Health and Its Relationship with the Prevalence of Obesity: An Infodemiological Study

**DOI:** 10.3390/nu15173773

**Published:** 2023-08-29

**Authors:** Liliana Melián-Fleitas, Álvaro Franco-Pérez, Javier Sanz-Valero, Carmina Wanden-Berghe

**Affiliations:** 1 Nutrition Department, University of Granada, 18012 Granada, Spain; lilianamelian@hotmail.es; 2 Geriatric Service, Insular Hospital, Health Services Management of the Health Area of Lanzarote, 35500 Arrecife, Spain; 3 Playa Blanca Health Center, Health Services Management of the Health Area of Lanzarote, 35580 Playa Blanca, Spain; 4 National School of Occupational Medicine, Carlos III Health Institute, 28029 Madrid, Spain; fj.sanz@isciii.es; 5 Health and Biomedical Research Institute of Alicante (ISABIAL), University General Hospital, 03010 Alicante, Spain; carminaw@telefonica.net

**Keywords:** obesity, diet, food, and nutrition, occupational health, health information, information search, infodemiology, Google Trends, body image

## Abstract

Objective: To identify and analyze population interest in obesity, nutrition, and occupational health and safety and its relationship with the worldwide prevalence of obesity through information search trends. Method: In this ecological study, data were obtained through online access to Google Trends using the topics “obesity”, “nutrition”, and “occupational health and safety”. Obesity data were obtained from the World Health Organization (WHO) website for crude adult prevalence and estimates by region. The variables studied were relative search volume (RSV), temporal evolution, milestone, trend, and seasonality. The temporal evolution of the search trends was examined by regression analysis (R2). To assess the relationship between quantitative variables, the Spearman correlation coefficient (Rho) was used. Seasonality was verified using the augmented Dickey–Fuller (ADF) test. Results: The RSV trends were as follows: obesity (R^2^ = 0.04, *p* = 0.004); nutrition (R^2^ = 0.42, *p* < 0.001); and occupational health and safety (R^2^ = 0.45, *p* < 0.001). The analysis of seasonality showed the absence of a temporal pattern (*p* < 0.05 for all terms). The associations between world obesity prevalence (WOP) and the different RSVs were as follows: WOP versus RSV obesity, Rho = −0.79, *p* = 0.003; WOP versus RSV nutrition, Rho = 0.57, *p* = 0.044; and WOP versus RSV occupational health and safety, Rho = −0.93, *p* = 0.001. Conclusions: Population interest in obesity continues to be a trend in countries with the highest prevalence, although there are clear signs popularity loss in favor of searches focused on possible solutions and treatments, with a notable increase in searches related to nutrition and diet. Despite the fact that most people spend a large part of their time in the workplace and that interventions including various strategies have been shown to be useful in combating overweight and obesity, there has been a decrease in the population’s interest in information related to obesity in the workplace. This information can be used as a guide for public health approaches to obesity and its relationship to nutrition and a healthy diet, approaches that are of equal utility and applicability in occupational health.

## 1. Introduction

Since the mid-1970s, the global prevalence of overweight and obesity has tripled; 2016 data indicated that more than 1900 million adults were overweight and, of these, more than 650 million were obese [1]. This increase has resulted from an inadequate diet (highly caloric and processed products), unhealthy lifestyles, and an increase in sedentary occupations, among other factors [2,3]. Obesity and its associated pathologies are related to greater morbidity and mortality, and a lower quality of life [3,4]. In addition, millions of dollars in costs and losses are generated for governments, society, and employers. In fact, productivity losses due to sick leave and presenteeism with emotional disengagement, are even greater than the direct costs of medical treatment [3,4,5].

In the era of big data and Web 2.0, such a widespread pathology with such far-reaching implications generates a constant flow of information and searches through networks, generating unprecedented opportunities for access to health information for patients and the general public [6,7]. In fact, the analysis of how people search and “navigate” the internet to obtain health-related information and how they communicate and share this information can provide valuable knowledge about the disease patterns, behavior, and health habits of populations [8,9,10]. Regarding the use of web-based data, it’s necessary to point out that the study of these new surveillance methods is in charge of infodemiology, defined by Eysenbach [9,11] as “the science of the distribution and determinants of information in an electronic medium, specifically the internet, or in a population, with the ultimate goal of informing public health and public policy”; i.e., to observe and analyze behavior from the internet to understand human behavior to then predict, evaluate, and even prevent health-related problems [12]. An early and well-known example of the use of internet data in health is the surveillance of influenza outbreaks, with an accuracy comparable to traditional methodologies [13].

Data sources relevant to infodemiology include Google Trends (GT) [14], a free online search tool that provides access to a sample of actual search requests made to Google, showing the interest users have had in a specific topic (globally or at the city level). It provides access to real-time data (a sample from the last 7 days) and non-real-time data (an independent sample of data from 2004 and up to 72 h before the search). Google Trends allows the users to define the search words as terms (obtains matches for the terms in the chosen language) or topics (a group of terms that share the same concept in any language) according to search parameters, and provides the geospatial and temporal patterns of search volumes for the terms specified. In addition, it is anonymous (no one is personally identified), categorized (based on the subject of search queries), and contains aggregated data (grouped) [12,14,15]. GT has been used to explore a wide range of topics in the health field [12] and has proven to be particularly useful in the early detection of health events such as diabetes [16] or obesity [17,18]. In addition, GT has also been shown to be very useful when it comes to studying population behaviors, and there are studies in the field of diet [19], occupational health [20,21], bariatric surgery [22], home care [23], gout [24], sexually transmitted diseases [7,25,26], as well as public health monitoring of disease outbreaks [27], among other research fields [28]. A review by Mavragani et al. [12] showed that these studies generally focus on examining seasonality, correlations, estimation models, and, to a lesser extent, provide predictions and forecasts. For further information on how Google Trends works, see https://support.google.com/trends and https://newsinitiative.withgoogle.com/google-news-lab.

As has been seen, population interest, observed through information search trends, can be useful as an indirect indicator that complements classic epidemiological indicators, since the data are easily manageable and at practically zero cost. In fact, there are already important studies demonstrating that search trends add value to traditional influenza surveillance systems [29] and even improve estimates of this disease [30]. Moreover, in other diseases, such as sexually transmitted diseases, a correlation between information search data and real disease data was apparent, indicating the importance of this type of association [7]. Likewise, the findings of Obeidat et al. [31] concluded that search queries were truly reliable in predicting disease outbreaks. Obviously, this study technique has also been tested in research on COVID-19, where Google searches for symptoms were found to predict actual increases in cases and hospitalizations during the pandemic [32]. At the same time, the research by Higgins et al. [33] confirmed the utility of digital epidemiology to provide useful surveillance data for disease outbreaks such as COVID-19. Although certain online search trends for this disease were influenced by media coverage, many search terms reflected clinical manifestations of the disease and showed strong correlations with real-world cases and deaths. Therefore, it can be stated, in line with Carneiro et al. [34], that this unique and innovative technology brings us one-step closer to true real-time outbreak surveillance.

Therefore, and as has been demonstrated in recent years, the study of information search trends can provide useful information about the interests of populations and population habits and behaviors [21,35]. Consequently, the objective of this work was to study and analyze population interest through information search trends on obesity, nutrition, and occupational health and safety and its relationship with data on obesity.

## 2. Materials and Methods

### 2.1. Design

This research constituted a descriptive, ecological, and correlational study. The methodological framework proposed by Mavragani & Ochoa [35] was followed to carry out the search strategies, as well as for data collection.

### 2.2. Source of Information

The information search data were obtained directly through GT (https://trends.google.com/trends/). Searches were carried out using the terms “obesity”, “nutrition”, and “occupational health and safety” as the subjects. Obesity data were obtained from the World Health Organization (WHO) website: Prevalence of obesity among adults, BMI ≥ 30, crude Estimates by WHO region (https://bit.ly/3PDFDpB).

The results obtained were downloaded in a standardized format (comma-separated values) that allowed their subsequent storage in an Excel file. Quality control of this information was carried out by means of double tables, correcting possible inconsistencies by consulting the original downloaded table.

The research data, as an open data source, may be freely used, reused, and redistributed and can be found using the following information:

Relative search volume data—DOI: https://doi.org/10.6084/m9.figshare.23256314

Search data by country—DOI: https://doi.org/10.6084/m9.figshare.23256323

### 2.3. Investigation Process

**Figure 1:**
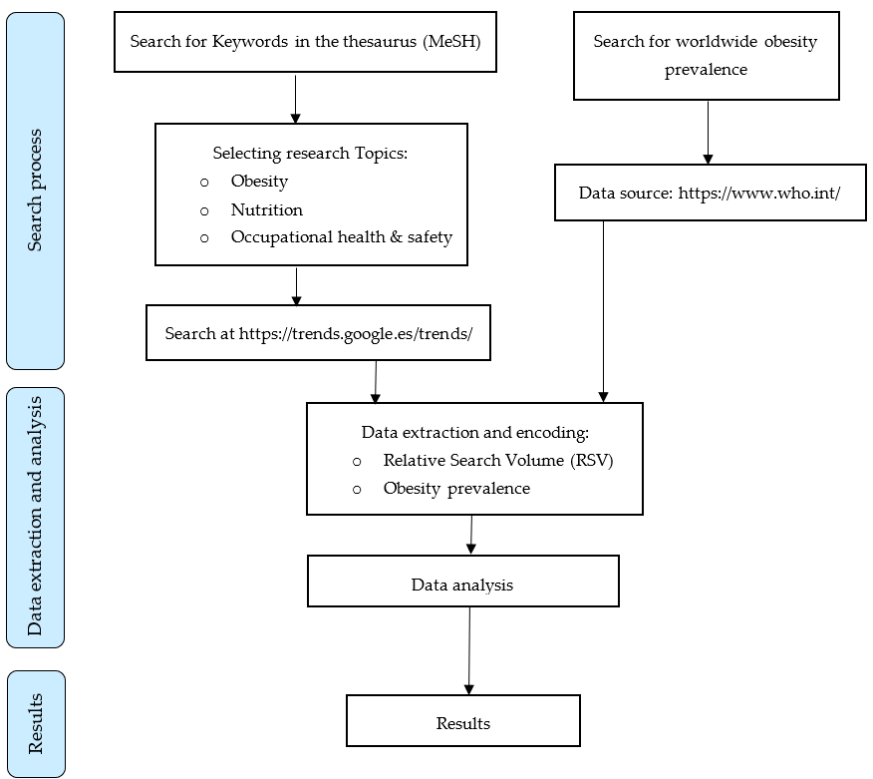
Flowchart explaining the research process.

The research process is shown in the following flowchart (Figure 1).

### 2.4. Variables under Study

The following variables were studied:

- Relative search volume (RSV): monthly result obtained through GT and normalized on a scale of 0 (RSV less than 1% of the volume) to 100 (maximum RSV). For example, an RSV of 25 indicates 25% of the highest observed search rate during the study period;
- Temporal evolution: long-term behaviors or trends for searches carried out on a specific topic;
- Milestone: one-off and prominent RSV event;
- Seasonality: periodic and predictable variation in a time series with a period less than or equal to one year.

### 2.5. Periods Analyzed

To analyze the RSV of the subjects under study, the period between 1 January 2004 (the first data provided by GT), and 31 December 2021, was analyzed. Annual data on the prevalence of obesity (total number of individuals in a population who have a disease or health condition at a specific period of time, usually expressed as a percentage of the population) [36] from 2004 to 2016 were obtained from the WHO; therefore, this period was used to correlate RSV with obesity data. The data retrieval date was 25 May 2022.

### 2.6. Data Analysis

Measures of central tendency were obtained to describe the following quantitative variables: mean and standard deviation (σ), median, interquartile range (IQR) and maximum and minimum. The temporal evolution of the search trends was examined by regression analysis, calculating the coefficient of determination (R^2^). To assess the relationship between quantitative variables, the Spearman correlation coefficient (Rho) was used. The level of significance used in all hypothesis tests was α ≤ 0.05. For this statistical analysis, the Statistical Package for the Social Sciences (SPSS) for Windows, version 28.0, was used.

Seasonality was verified using the augmented Dickey–Fuller (ADF) test. The unit root test was carried out under the null hypothesis α = 0 against the alternative hypothesis of α < 0. This analysis was performed with R version 4.0.3 (*p* > 0.05 indicated a significant statistical result for the ADF test).

### 2.7. Related Queries

The list of terms used in the searches allowed us to identify the different ways used by the population to obtain information about the topics under study.

If searches are performed in GT using the term “Topic”, GT will display the matches of all the query terms in the specified language (for example, if “Health” is searched, results will also be obtained for “public health”, “health science”, “occupational health”, etc.). However, in addition, the use of the word “Topic” will yield results for terms that share the same concept in any language (for example, if “Health” is searched, the results will include terms such as “health”, “salut”, “saúde”, “safety”, and “work safety”, among others) [37]. Therefore, a search by topic encompasses related terms. This accumulation of terms is known as Long Tail and reflects the informational set of the community.

Because the search by topic encompasses related queries, it can identify the accumulated interest of the population for certain information because the search with technical words is not usually frequent in GT [38]. In this study, the related terms also pertained to those studied so that the RSV was not influenced (the main terms included the related terms).

## 3. Results

Using GT, RSVs were obtained for the topics under study (“obesity”, “nutrition”, and “occupational health and safety”), and obesity prevalence data were obtained from the WHO website. The annual data are reported in Table 1.

The central tendency statistics for RSV are provided in Table 2.

**Figure 2:**
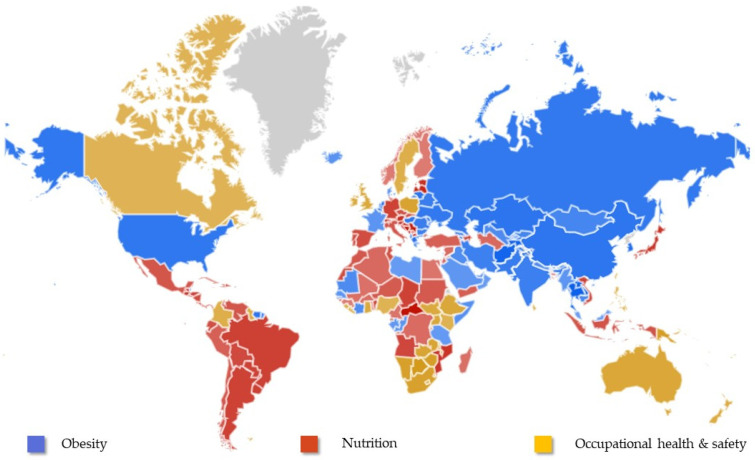
Comparative breakdown by country for the global results for the topics of obesity, nutrition, and occupational health and safety (from 1 January 2004 to 31 December 2021), obtained from Google Trends (www.google.com/trends).

Taking the data and images provided by GT, global RSVs were obtained, and main interests were observed by country; see Figure 2 (color intensity represents the percentage of searches, and gray indicates a lack of data for that area).

### 3.1. Related Queries

The searches, with other terms, carried out by users who searched for the topics under study, ordered based on percentage in relation to the main topic, are reported in Table 3.

### 3.2. Temporal Evolution of RSVs

**Figure 3:**
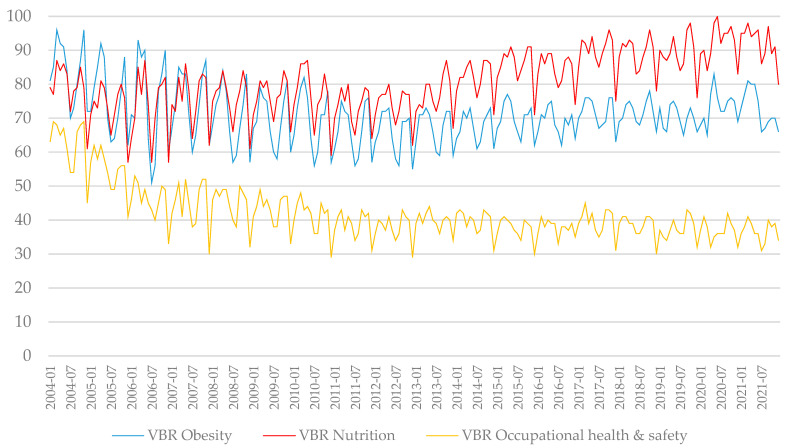
Search trends, obtained from Google Trends, for the topics of obesity, nutrition, and occupational health and safety (from 1 January 2004, to 31 December 2021).

From the relative search volume (RSV) data provided by GT, a graph of the temporal evolution of the results was constructed for the terms under study (see Figure 3).

The annual RSV for obesity showed a very low decreasing linear trend (R^2^ = 0.04, *p* = 0.004); for nutrition, the observed trend was linear, with moderate growth (R^2^ = 0.42, *p* < 0.001); and for occupational health and safety, there was an exponential moderate decreasing trend (R^2^ = 0.45, *p* < 0.001).

For the annual data on obesity provided by the WHO, a very strong exponential trend was observed (R^2^ = 0.99, *p* < 0.001).

### 3.3. Main Milestones

The main RSV events for obesity occurred worldwide in March 2004 (RSV = 96); for nutrition, the main RSV events occurred in June 2020 (RSV = 100, the maximum possible value); and for occupational health and safety, the main RSV events occurred in November 2011 (RSV = 69); see Figure 3.

The valleys observed in Figure 3 generally occurred in December for the three topics; in contrast, the peaks occurred in different months over time.

### 3.4. Seasonality

The analysis of seasonality demonstrated the absence of a periodic and predictable temporal pattern for each term studied: ADF obesity, −4.06; ADF nutrition, −4.09; and ADF occupational health and safety, −3.97, with *p* values < 0.05 for all three terms.

### 3.5. Relationship between the Prevalence of Obesity and the RSV Studied

**Figure 4:**
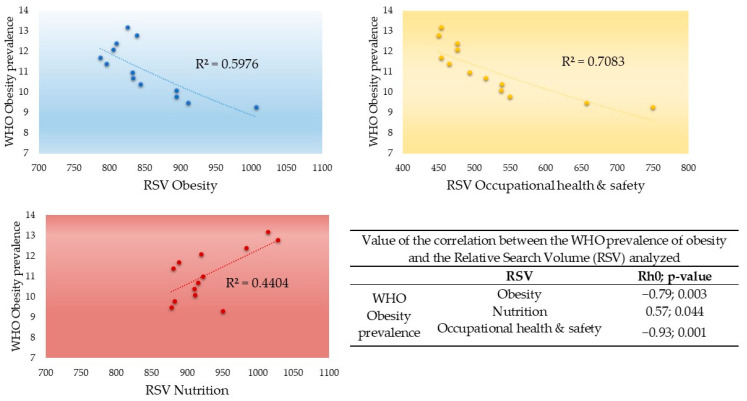
Correlations between the annual prevalence of obesity (WHO) and the different relative search volumes (RSVs) studied.

The correlation analysis of the obesity data provided by the WHO and the RSVs of the three subjects under study revealed the existence of a significant indirect association between the prevalence of obesity and the RSVs for obesity and occupational health and safety. Additionally, there was a significant direct association between the prevalence of obesity and the RSV for nutrition. A graphical representation of the relationships between the prevalence of obesity and the RSVs studied and the correlation values (Rho) can be found in Figure 4.

## 4. Discussion

The results obtained suggest that trends from search engines may be a tool capable of identifying, in real time, the information needs of the population in relation to the subjects studied. In 2014, Anderegg & Goldsmith [39] affirmed that GT can be used as a solid and valid tool for predicting behavior patterns in the search for information. Likewise, Gizzi et al. [40] showed that monitoring online behavior by GT can be useful for stakeholders and policymakers to put into the field well-timed and geographically-targeted information and communication action plans. This information could be used to implement timely and geographically-targeted food education and health education campaigns based on the use of real-time data derived from GT and the related Geomaps that show where increased population interest is taking place.

The area of health, both nutritional and occupational health, is rich in data and information, and search engines provide the possibility of managing data generated by users in real time [41]. The use of this potential, as has been seen in this work, made it possible to assess the health information needs of a population. In this ecological and infodemiological study, the global popularity of searches related to obesity, nutrition, and occupational health and safety and their relationships with obesity prevalence were studied. The analysis revealed some results that warrant detailed discussion.

Although the global data pointed to greater population interest in information on nutrition over obesity and occupational health and safety, this result was not the same for all countries and varied by demographic factors and epidemiological characteristics of each area. The nutritional transition is not a simple and obvious replacement for eating habits and lifestyle, but rather a complex process where multiple causes converge [42]. What is already clear is that obesity and overweight are related, in part, to working conditions. In addition, obesity can increase the risk of occupational diseases and injuries [43].

Regarding searches by country, notable differences were observed. In the USA, the main search topic was obesity, a finding that is not surprising because according to data from the National Health and Nutrition Examination Survey (NHANES), more than 40% of American adults are obese [44]; additionally, in relation to the number of inhabitants, the USA has the highest prevalence of obesity worldwide, without taking into account, of course, the extreme cases of Pacific islands such as Tonga, Samoa, and Niue [1]. Although obesity is also linked to poverty, high- and middle-income countries stood out in searches for obesity.

In Latin America and the Caribbean, the volume of searches on nutrition could be explained by the concern for correcting the undernourishment of this population, as this is the only region in the world to achieve the goal set by the Millennium Development Goals of reducing by half the percentage of people who suffer from hunger [45].

Most Mediterranean countries showed greater interest in information on nutrition. The dietary model with the greatest evidence of health benefits is that traditionally followed by the inhabitants of some Mediterranean countries [46], and consequently, this information-seeking behavior is not surprising.

The fact that occupational safety and health was the most sought-after topic in Canada could be related to the strong awareness that workers have about the ways in which work could damage their health, as indicated by Walters & Haines in their 1988 study [47]. This study also indicated that health and safety concerns would be eased if workers had better access to information on their labor rights and mechanisms to deal with hazards in the workplace. A similar situation was observed for Australia, as the increasing popularity of occupational safety and health management systems has stimulated a critical debate on their effectiveness [48]. The opposite results were observed for searches in the Horn of Africa, where not only job security is in question, but sometimes human rights are compromised [49].

What has been identified in this section has been the main interest of the population when seeking information on obesity, nutrition, and occupational health.

### 4.1. Temporal Evolution of RSVs

The temporal evolution of the RSV for nutrition indicated increasing interest over time, either as a health trend or as a lifestyle, mainly in countries with higher purchasing power. Additionally, this topic was notably influenced by the related queries, where among others, searches on vegan, vegetarian, or gluten-free diets were popular [50].

In contrast, the RSV for occupational health and safety exhibited a decreasing trend which has already been identified and discussed in a previous study [21]. A similar decreasing trend was observed for obesity, also evidenced in previous studies [17,18].

The loss of interest observed regarding occupational health and safety is worrying because the workplace and all the factors that derive from it (shifts, stress, rest, availability of food, etc.) can promote obesogenic environments and habits. In this sense, multiple studies and organizations have demonstrated the importance of the workplace and its relationship with food and diet as a fundamental vector for the prevention of overweight and obesity [51,52].

More worrying, if possible, is the perception of the population regarding obesity, which has turned toward “normalization”. Thus, health surveys among the obese and overweight population show that a significant number of these individuals perceive their weight as “normal” [53]. This alarming fact demonstrates that the public concept of obesity can be influenced by the continuous increase in its prevalence. Likewise, since 2013, an increase has been detected in searches for the terms “body positivity” and “self-love”, which suggests a deviation in the public interest in obesity, more related to image than to implications for health [18]. An example of this is the powerful movements in social networks towards acceptance of fat, created in a reactive way to the idealization of the slim body as an ideal of health and aesthetics, especially for women [54,55].

At present, the population has become accustomed to living with obesity on a daily basis, and evidence of this is the worldwide prevalence data which marks an ascending and unceasing increase since the end of the last century [1]. The paradox is that the opposite occurs when analyzing the interest of the population in information on the internet related to obesity. An inverse relationship was observed between the prevalence of obesity and the RSV, a finding that is consistent with the results of previous studies [17,18,56]. This apparent disinterest in the population regarding obesity is discouraging considering the enormous costs (social, labor, economic, and health) derived from it and its comorbidities [3,5].

### 4.2. Milestones

No clear and specific fact was found for any of the subjects studied with which to relate them. The temporal evolution presented a sawtooth pattern in which no milestone of special interest could be highlighted. The appearance of search peaks (milestones) provides important information for epidemiological surveillance, as has already been demonstrated for some diseases [27], either due to relevant outbreaks or in response to specific advertising campaigns that translate into greater population interest (reflected in the increase in information searches) [23].

Milestones are usually reached approximately two weeks after shocking news related to health is published, and therefore, it is difficult to place them on a timeline [57]. Additionally, peaks in search volume are difficult to interpret but help improve epidemiological surveillance [34].

The sawtooth pattern allows graphical recognition of the relationship between turns of a dialog, a typology of interactional figures representing a collection of different structures that occurs in the turn-taking of colloquial conversation (verbal, written, or digital) [48].

For obesity, there was a lack of a clear milestone, potentially because until 2020, World Obesity Day (celebrated annually since then, on March 4) and the associated website [58] had not been well promoted. Previously, advertising against obesity was designed from the perspective of diabetes [59]. The other two subjects under study could not be found to be related to any event with a global impact.

### 4.3. Seasonality

Seasonality is a concept frequently used in public health studies. It assumes that health-related variables undergo regular fluctuations or changes over time making them predictable and facilitating their temporary study and, of course, prevention. The importance of measuring seasonality is associated with improvements in prognosis and prevention, and is valuable to adapt goods and services based on demand [60].

In this study, no seasonality was observed for any of the three subjects studied, and the existence of a periodic temporal pattern could not be demonstrated during each of the years analyzed, indicating that the searches carried out by the population do not have an “expected” trend related to certain times of the year. As Kardeş [24] indicated, to estimate seasonal variations in internet searches, more consistent data are necessary to elucidate the mechanisms that establish such seasonality.

The monitoring of online consultations is more valuable when there are changes in behavior [12], and in areas such as public health, changes can represent a new source of data informing about the health of the population. Although they are not currently taken into account by epidemiological surveillance models, this information can be used in a complementary way alongside surveillance systems [7]. As Carneiro & Mylonakis [34] state, this unique and innovative technology may provide a closer step toward achieving true real-time health surveillance.

### 4.4. Relationship between the Prevalence of Obesity and the RSVs Studied

Despite a growth in the global prevalence of obesity, an inverse association was found with population interest, as shown through information searches on issues related to obesity itself. In contrast, the interest in information related to nutrition experienced significant growth—a direct relationship—that was associated with the increase in the prevalence of obesity, indicating that the focus shifted from searches related to the pathology to possible solutions. That is, searches focused on treatments, healthy habits and behaviors aimed at solving obesity and overweight gained popularity. Proof of this is the boom in searches for terms related to exercise [6], weight loss [18], diet [19], and bariatric surgery [61]. The increases in searches for these terms are also equally valid and accurate indicators of changes in the prevalence of obesity [6]. Moreover, Wang & Chen’s [62] findings indicated that when the economy is in recession, people tend to search less for information related to obesity and health behaviors; however, they are more likely to search for fast food restaurants.

Regarding occupational health, although the relationship between obesity and long working hours, shift rotation, night work, and high work stress has been demonstrated [63], similar relationships were not observed between workers’ prevalence of obesity and interest in information on occupational health. This inverse relationship indicates that although most people spend a large part of their time in the workplace (and therefore eat at least one of their daily meals there) and that well-planned interventions—preferably including several strategies—were shown to be useful to combat overweight and obesity [51,52], this fact was not reflected in the population’s interest in seeking existing, related information.

### 4.5. Limitations

Search engine trends serve as a tool that can identify, both through real-time surveillance and temporal analysis (periods), the needs and interests of the population in relation to health information (and information related to other fields) [12,28]. In this regard, it is important to note that this study encompasses an analysis of ecological data and, therefore, the findings may not be representative at the individual level, both owing to the study design and the tool’s limitations [64]. Likewise, this study is limited to the “connected world” (the so-called digital divide), and therefore, there is possible bias regarding the results that can be extracted from the behavior patterns of the population [6,12].

In the same way, it should be noted that this study was based solely on searches carried out through Google (without taking into account other search engines), however, this browser had a market greater than 92% in 2021 [65]. Another limitation of the study is that it has focused on the global prevalence of obesity in adults, however, GT searches cannot be filtered by sociodemographic factors, so it is plausible that a percentage of these searches have been carried out by non-adults. Similarly, and because the search sample is unknown, other demographic factors such as age and sex cannot be included in the analysis [35].

As noted by Johnson and Mehta [25], a possible drawback of GT is that it does not provide real-use data and precise time intervals, thus reducing the forecasting capacity. In addition, there is a lack of complete transparency because there is no information on the specific methods and models that Google uses to calculate RSV; additionally, as has been suggested in several publications [21,26,35,66], the results obtained with this tool could be influenced by the interest of the media, mainly advertising campaigns, which may not correspond exactly to the interest of the general population.

Another well-known bias is that a part of Google Trends data can also reflect irregular search activity, such as automated searches or queries that may be associated with attempts to spam the Google search results. Given this, users should understand that the data is not a perfect mirror of search activity. Besides, GT does filter out searches made by very few people, showing only data for popular terms, so search terms with low volume appear as “0” [15].

The lack of research in the field of infodemiology, despite being a rapidly growing area of knowledge, means that these results cannot replace conventional surveillance systems. Even so, they can represent a new source of data on the health of the population and be useful as a complement to traditional systems [17,50]. Further studies on the utility and limitations of these methodologies are necessary.

Although it is true that healthy lifestyles are key in interventions related to nutritional disorders [11], in the studies on RSV we obtained data on this trend that suggests the population’s need for information, being an ecological study; however, we cannot know the causes that triggered the search for information.

Finally, and although the objective of our study was focused on nutrition and occupational health, we cannot forget that obesity rates are highly influenced by lifestyle choices, such as dietary factors, physical activity, sedentariness, sleep patterns, genetics and gene–lifestyle interactions, environmental exposures, and built environments, among others [67]. Future research could focus on these issues.

## 5. Conclusions

Population interest in obesity continues to be a trend in the countries with the highest obesity prevalence, although there are clear signs of loss of popularity in favor of searches focused on possible solutions and treatments, with a notable increase in searches related to nutrition and diet.

Despite the fact that most people spend a large part of their time in the workplace, and that various intervention strategies were shown to be useful to combat overweight and obesity, there was a decrease in the population’s interest in information related to obesity in the workplace.

The incessant growth in obesity worldwide and the changes in behavior and perception about it, forces different actors in the field of health to address this problem in multiple ways. With knowledge of the interests of the population, obtained through search trends, it is essential to promote prevention and intervention programs, for example, and to consider the new phenomena related to body positivity and the normalization of obesity.

This information can be used as a guide for policymakers as well as public health approaches to obesity and its relationship to nutrition and a healthy diet, approaches that are equally useful and applicable in occupational health.

The findings of this study should encourage further research on how infodemiology and Big Data can help society, companies, and governments to guide policies and educational campaigns to address the growing obesity epidemic, reaching population niches through new technologies and acting on population interests and threats.

## Figures and Tables

**Table 1:** Data grouped by year for relative search volume (RSV) for obesity, nutrition, and occupational health and safety and the worldwide prevalence of obesity.

Year	RSV Obesity	RSV Nutrition	RSV Occupational Health and Safety	World Obesity Prevalence
2004	1007	950	749	9.30
2005	911	878	657	9.50
2006	894	882	549	9.80
2007	894	911	537	10.10
2008	844	910	538	10.40
2009	833	915	516	10.70
2010	832	922	493	11.00
2011	796	880	465	11.40
2014	787	888	453	11.70
2015	805	920	476	12.10
2016	810	983	476	12.40

**Table 2:** Statistics, for the entire period analyzed, for the relative search volume (RSV) for obesity, nutrition, and occupational health and safety.

Topic	Mean ± σ	Median	AIQ	Maximum	Minimum
Obesity	71.24 ± 0.57	71	9	96	51
Nutrition	81.00 ± 0.62	81	13	100	57
Occupational health and safety	42.02 ± 0.54	40	8	69	29

**Table 3:** Terms used by users to perform searches related to the topics of obesity, nutrition, and occupational health and safety and the percentage of each in relation to the main topic *.

Obesity		Nutrition		Occupational Health and Safety	
Terms	%	Terms	%	Terms	%
Obesity	100	Nutrition	100	Health	100
Obese	55	Nutriçao	38	Health Safety	98
Overweight	39	Beslemne	30	Safety	96
Obesidade	16	Nutricionista	29	Salud	31
BMI	10	Valori nutrizionali	13	Occupational health	28
Diabetes	7	Nutrisi	11	Work safety	14

* Spelling accents were not taken into account in the related terms because Google does not report whether or not they were used at the time of the search.

## Data Availability

Data supporting reported results can be found at: Relative search volume data—DOI: https://doi.org/10.6084/m9.figshare.23256314. Search data by country—DOI: https://doi.org/10.6084/m9.figshare.23256323.
